# Functional and pathologic association of aminoacyl-tRNA synthetases with cancer

**DOI:** 10.1038/s12276-022-00765-5

**Published:** 2022-05-02

**Authors:** Yulseung Sung, Ina Yoon, Jung Min Han, Sunghoon Kim

**Affiliations:** 1 Yonsei Institute of Pharmaceutical Sciences, College of Pharmacy, Yonsei University, Incheon, 21983 South Korea; 2 Institute for Artificial Intelligence and Biomedical Research, Medicinal Bioconvergence Research Center, College of Pharmacy and College of Medicine, Gangnam Severance Hospital, Yonsei University, Incheon, 21983 South Korea; 3 Department of Integrated OMICS for Biomedical Science, Yonsei University, Seoul, 03722 South Korea

**Keywords:** Mechanisms of disease, Nutrient signalling, Phosphorylation, Cancer metabolism, Cancer genomics

## Abstract

Although key tumorigenic and tumor-suppressive factors have been unveiled over the last several decades, cancer remains the most life-threatening disease. Multiomic analyses of patient samples and an in-depth understanding of tumorigenic processes have rapidly revealed unexpected pathologic associations of new cellular factors previously overlooked in cancer biology. In this regard, the newly discovered activities of human aminoacyl-tRNA synthases (ARSs) deserve attention not only for their pathological significance in tumorigenesis but also regarding diagnostic and therapeutic implications. ARSs are not only essential enzymes covalently linking substrate amino acids to cognate tRNAs for protein synthesis but also function as regulators of cellular processes by sensing different cellular conditions. With their catalytic role in protein synthesis and their regulatory role in homeostasis, functional alterations or dysregulation of ARSs might be pathologically associated with tumorigenesis. This review focuses on the potential implications of ARS genes and proteins in different aspects of cancer based on various bioinformatic analyses and experimental data. We also review their diverse activities involving extracellular secretion, protein–protein interactions, and amino acid sensing, which are related to cancers. The newly discovered cancer-related activities of ARSs are expected to provide new opportunities for detecting, preventing and curing cancers.

## Cancer: A role for some of life’s most crucial enzymes

Enzymes called aminoacyl-tRNA synthetases (ARSs), which play a central role in all life, are becoming implicated in several aspects of cancer in ways that may lead to new approaches for prevention, detection and treatment. ARS enzymes catalyse the ligation of amino acids to transfer RNA molecules to allow amino acids to combine in the correct sequences to form proteins. Jung Min Han, Sunghoon Kim and colleagues at Yonsei University, Incheon, South Korea, review researches implicating ARS enzymes and the genes that code for them in a variety of cancers. The behavior of ARS enzymes and their genes are found to be altered in several types of cancer cells in ways that may either initiate or support the onset and development of the disease, through which they could be suggested as targets for novel anti-cancer drugs.

## Introduction

**Fig. 1:**
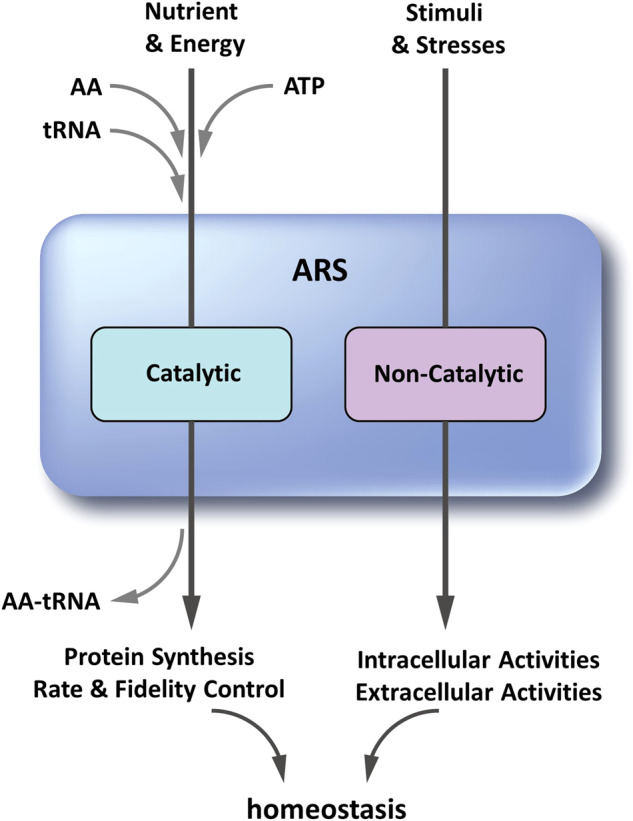
Dual activities of ARSs for system homeostasis. The catalytic and noncatalytic functions of ARSs are important for homeostasis. The catalytic function of ARS is to produce aminoacyl-tRNAs from amino acids and ATP for protein synthesis. Their catalytic activities can be controlled to adjust the protein synthesis rate and fidelity in coordination with nutrients (particularly amino acids) and energy status. In another axis, ARSs can sense various stimuli and stresses and mediate cellular responses via unique extracellular and intracellular activities. These two lines of activities cooperatively function in system homeostasis.

The well-known function of aminoacyl-tRNA synthetases (ARSs) is to match specific amino acids to their cognate tRNAs and covalently link them for protein synthesis (Fig. 1, left axis)^1^. For this reason, most human diseases associated with mutation or aberrant expression of ARSs have been investigated from the point of their roles in translation. However, rapidly accumulating evidence shows that ARSs have evolved to play diverse and crucial roles in system development and homeostasis (Fig. 1, right axis). These new findings suggest that etiological investigation of ARS-associated diseases needs to be considered not only with respect to the catalytic activities of these enzymes for translation but also their noncatalytic roles beyond this process.

Recent genomic, proteomic and bioinformatic analyses have unveiled a pathologic link between human ARSs and various cancers as well as other human diseases. Through unique additional domains, such as WHEP, leucine zipper, and alpha-helices^2–6^, ARSs can pathologically participate in sustaining proliferative signals, deregulating cellular energetics, financing tumor-promoting inflammation, and promoting metastasis and angiogenesis. Furthermore, cancer-associated gene expression, mutation, and structural modification of ARSs might be related to their multifunctional properties^7^. An irregular event in ARS transcription and translation can result in unrestrained cellular signals that contribute to tumorigenesis. In addition, ARS gene expression patterns can be used as a possible biomarker for cancer^8^. Overall, genetic and postgenetic abnormalities of human ARSs appear to be deeply linked to cancer development and survival through nonconventional and catalytic activities.

## Cancer-associated expression of ARSs

In this section, different ARS expression patterns shown in different cancer types are discussed. Transcriptional and posttranscriptional regulation are also reviewed as factors influencing ARS expression patterns.

### Transcription

**Fig. 2:**
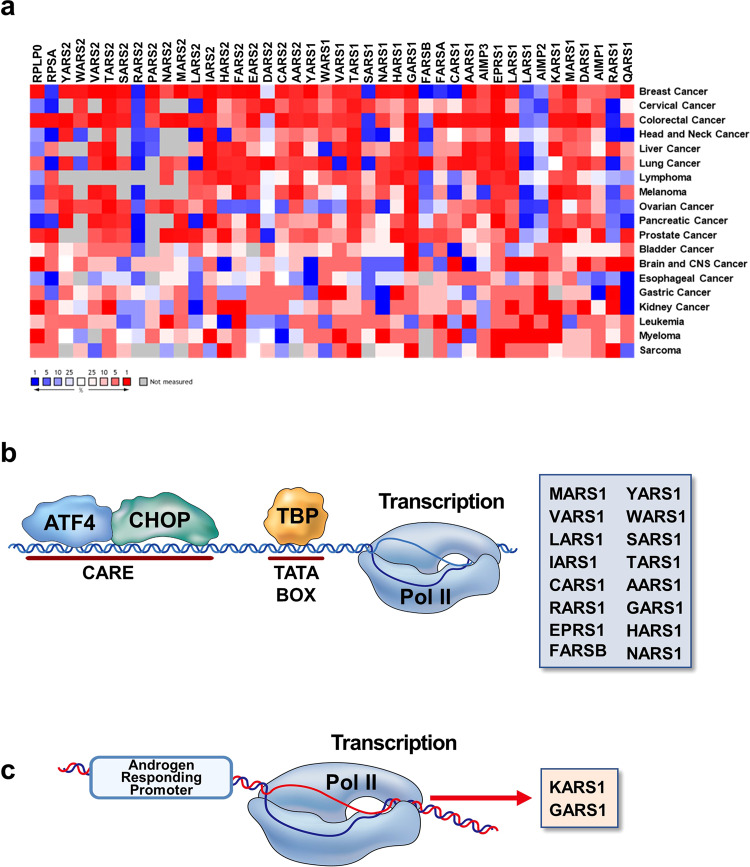
ARS gene expression in cancer. **a** Heatmap of ARS and ribosomal subunit gene expression in different cancers. Gene expression information was obtained from TCGA and analyzed using Oncomine Data Tools. The gene expression data were extracted by ordering the genes according to the *p* values from the whole gene pool across the respective analysis. The corresponding median gene ranks are displayed in a color gradient from overexpression (red) to underexpression (blue). No cancer-dependent expression change is indicated with white color. XARS, aminoacyl-tRNA synthetase for amino acid “X”, 1 and 2 represent the cytosolic and mitochondrial forms, respectively. **b** ATF4-mediated transcriptional control of ARSs. Interaction of CARE with ATF4 constitutes the initiating step of ARS gene expression, followed by assembly of CHOP and TBP. When the complete transcription machinery has assembled, RNA polymerase II is recruited and initiates ARS gene expression. **c** Promoters with androgen response elements are involved in *KARS1* and *GARS1* transcription, which may be involved in endocrine cancers.

Alteration of gene expression patterns is a key property of cancer, which might be either a cause or effect of cancer, providing both a tumor-friendly environment and inducing stress responses. Changes in the expression patterns of the genes encoding 37 ARSs and 3 AIMPs (20 cytosolic and 17 mitochondrial ARSs and AIMP (ARS-interacting multifunctional protein) 1, 2, 3) were examined in 19 different cancer types from the open database TCGA, as depicted as a heatmap in Fig. 2a. If ARSs are dedicated only to catalytic activities for translation, their expression is expected to be generally upregulated in cancer cells to meet the increased demand for protein synthesis. However, the heatmap shows no general cancer-associated gene expression pattern among ARS genes but rather unique expression patterns depending on ARSs and cancer types.

First, there are significantly overexpressed (1% and lower) ARSs, as supported by experimental data. For instance, EPRS1, LARS1, MARS1, TARS2, and YARS2 show increased gene expression in pancreatic adenocarcinoma, clear cell renal cell carcinoma, prostate adenocarcinoma, diffuse large B cell lymphoma, and follicular variant of papillary thyroid carcinoma, respectively^9–13^. In contrast, LARS1, NARS1, QARS1, and LARS2 are significantly underexpressed in acute myeloid leukemia, pancreatic adenocarcinoma, squamous cell lung carcinoma, and clear cell renal cell carcinoma, respectively. The idiosyncratic expression pattern of ARSs in different cancers suggests unique roles for individual ARSs in cancer biology while their catalytic activities are canonically required for protein synthesis in cancer cells. To validate whether changes in ARS gene expression are pathologically significant, further studies are necessary to examine whether such gene expression changes are also reflected at the protein level and mechanistically related to cancer progression or suppression.

### Transcriptional regulation of cancer-related ARSs

Expression of the ARS gene family is induced in response to amino acid limitation by activating transcription factor 4 (ATF4)^14,15^. ATF4 is a stress-induced transcription factor that is commonly upregulated in cancer cells^14^. Most cytosolic ARS-encoding genes (16 of 20; *AARS1, CARS1, EPRS1, FARSB, GARS1, HARS1, IARS1, LARS1, MARS1, NARS1, RARS1, SARS1, TARS1, VARS1, WARS1* and *YARS1*) are transcriptionally induced by ATF4. Mechanistically, transcription of 16 ARS-encoding genes is initiated by C/ebp-Atf response element (CARE) enhancers^14^. ATF4 and C/EBP-homologous protein (CHOP) bind to CARE, and TATA-binding protein (TBP) is recruited to the TATA box^16^ (Fig. 2b). Following complete formation of the transcription machinery, RNA polymerase II initiates transcription of ARS genes. Considering that ATF4 expression is increased under oxidative stress, ER stress and hypoxia, as well as amino acid deprivation, ARS gene transcription can be changed under tumorigenic conditions^17^.

Another well-known ARS transcription-inducing promoter is the androgen response element (ARE)^12^. Hormonal receptor alteration is one of the main characteristics of endocrine cancers^18^. GARS1 and KARS1 display androgen-dependent transcriptional initiation in several hormone-responsive cells, such as prostate cancer cells (Fig. 2c)^19^; thus, transcription of GARS1 and KARS1 is initiated in cancers with increases in AREs. The importance of EPRS1 expression in estrogen receptor-positive breast cancer has also been monitored in clinical samples^20,21^.

### Post-transcriptional control of ARSs

Posttranscriptional regulation of ARSs, including alternative splicing and alternative polyadenylation, is also associated with tumorigenesis. Alternative splicing occurs in the 5ʹ untranslated region (UTR) of *WARS1*, producing *exon II-lacking WARS1* (*mini-WARS1)* mRNA^22^. In contrast to full-length WARS1, mini-WARS1 inhibits vascular endothelial growth factor (VEGF)-induced angiogenesis, which is crucial for a steady supply of nutrients to tumors^23,24^.

Second, *CARS1* is also subjected to a unique splicing mechanism, generating the variant CARS1-N6, in which a specific sequence is inserted at the N-terminal GST domain^25^. This variant inhibits eukaryotic translation elongation factor 1 gamma (EEF1G), which is known to be overexpressed in various cancers, such as esophageal carcinoma, pancreatic cancer, and adenocarcinoma of the colon^26–28^, suggesting a potential role for CARS1 variants in cancer through association with EEF1G.

An alternative polyadenylation-mediated variant of EPRS1, EPRS1-N1, lacking the PARS1 part in the *EPRS1* transcript is found in various human cell lines^29^. Alternative polyadenylation of the *EPRS1* transcript starts at the 864^th^ codon and changes UAU to UAA, leading to an incomplete EPRS1 protein. In response to IFN-γ, full-length EPRS1 forms an IFN-γ-activated inhibitor of translation (GAIT) complex along with NS1-associated protein 1 (NSAP1), ribosomal protein L13a (L13a), and glyceraldehyde-3-phosphate dehydrogenase (GAPDH) to inhibit translation of specific target mRNAs (GAIT element RNAs) in monocytic cells^6^. Although EPRS1-N1 also interacts with GAIT-element RNAs, such as VEGFA transcripts, it blocks translational repression of the GAIT complex. Considering the significance of immune cell-mediated microenvironment changes in cancer development, modulating the function of the GAIT complex through this variant may also affect cancer^29–31^. Understanding the pathologic association of these ARS variants would provide insight into the working mechanisms of their native forms related to malignant cell transformation and cancer development.

## ARS gene alteration in cancer

Below, ARS gene alterations, including single-nucleotide polymorphisms (SNPs), genetic mutations, and chromosomal rearrangements, are reviewed (Table 1).

**Table 1:** ARS gene alteration in cancer.

Alteration Type	ARS	Detail	Affected Region	Related Cancer Type
SNP	DARS1	G/T (rs3768998)	Aspartate/asparagine metabolism	Either B-Lymphoblastoid Cell Lines or Acute Lymphoblastic Leukemia
		T/C (rs7587285)		
		T/C (rs11893318)		
		T/C (rs2322725)		
		A/C (rs2278683)		
	NARS1	C/T (rs2318301)		
		C/G (rs540680)		
	DARS2	A/G (rs2068871)		
		C/T (rs2759328)		
		C/T (rs941988)		
		C/T (rs2227589)		
		A/G (rs16846526)		
	NARS2	G/A (rs11237537)		
	AARS1	G/A (rs34087264)	5ʹ cis-eQTL^a^	Breast Cancer
	HARS1	A/G (rs801186)	Intron 11	
	RARS1	A/G (rs193466)	Intron 1	
	WARS1	A/G (rs2273802)	5ʹ UTR	
	CARS1	A/C (rs384490)	Transcription factor binding site	Gastric Cancer
		A/G (rs729662)	Exon splicing enhancer	
		G/A (rs2071101)	DNase I Binding Site	
		A/G (rs7394702)	DNase I Binding Site	
Point Mutation	IARS2	A – Deletion	Promoter	Nonpolypopsis Colorectal Cancer, Turcot Syndrome
		TA – Deletion		
	RARS1	miR-15	Locus 13q14	Chronic Lymphocytic Leukemia, Pituitary Adenoma
	RARS1	miR-16	Locus 13q15	
Frameshift Mutation	MARS1	Premature Stop Codon (Loss of Catalytic Domain)	Exon 3 T9 repeat	Colorectal Cancer, Gastric Cancer
Chromosome Rearrangement	CARS1	CARS1-ALK fusion	t(2;11;2) (p23;p15;q31)	Inflammatory Myofibroblastic Tumor
	MARS1	TLS-CHOP stabilization	t(12;16)(q13;p11)	Myxoid and Round Cell Liposarcomas
Alternative polyadenylation	EPRS1	Y864 into Stop Codon	Y864	Monocyte Cancer
Deletion	LARS2	Entire LARS1 including chromosome loss	3p21.3	Nasopharyngeal Carcinoma
	CARS1	Entire CARS1 including chromosome loss	11p15.5-p15.4	Wilms Tumor and Embryonal Rhabdomyosarcoma, Adrenocortical Carcinoma, and Lung, Ovarian and Breast Cancer

ARS gene alterations include SNPs, mutations, and chromosomal rearrangements, and they are uniquely associated with different types of cancer.

^a^eQTL: expression quantitative trait locus.

### Cancer-associated SNPs

SNPs in some ARSs are associated with cancer risk or with sensitivity to anticancer therapy. SNPs in the *DARS1, NARS1, DARS2* and *NARS2* genes are reportedly related to cancer. These SNPs were detected in either B-lymphoblastoid cell lines or primary cells of acute lymphoblastic leukemia (ALL) (Table 1).^32^ Cells with these SNPs exhibit altered sensitivity to asparaginase treatment, possibly due to changes in aspartate/asparagine metabolism^32^. Considering the importance of aspartate/asparagine metabolism for the development of cancer, including ALL, these SNPs might play a role in tumorigenesis as well as in sensitivity to anticancer therapy^33^.

Through a case–control study of breast cancer in the Chinese population, SNPs in *AARS1, HARS1, RARS1* and *WARS1* were found to be associated with an increased risk of breast cancer (Table 1)^34^. Considering that these SNPs are observed in 5ʹ UTRs or introns, they might affect cancer risk by changing gene expression levels. Another study suggested the association of SNPs in *CARS1* with an increased risk of gastric cancer in the Chinese population^35^. Through a two-stage case–control study, four SNPs in *CARS1* were found to be potentially functional (Table 1). Using the Santa Cruz Genome Browser website and some other web-based analysis tools, the authors predicted that these SNPs disrupt transcription factor response elements or DNA methylation levels, affecting *CARS1* expression levels^35^.

### Genetic mutations

Several point mutations and frameshift mutations in ARS genes are found at considerable frequencies in some types of cancer, with implications for tumorigenesis (Table 1). For example, A or TA deletion in the promoter region of *IARS2* is reported in 59% of nonpolyposis colorectal cancer and Turcot syndrome. The (A)_10_(TA)_9_ repeat is normally observed in the 5ʹ upstream position of the *IARS2* gene; in the −318~−291 position, alterations of (A)_9_(TA)_8_ or absolute deletion of the wild-type allele are frequently observed in tumors. Although the effects of these mutations on IARS2 expression are not yet understood, TA repeat deletion may result in its underexpression^36^. A frameshift mutation in *MARS1* has been reported in colorectal or gastric cancer, and a frameshift caused by deletion of a T in the T9 repeat sequence in exon 3 results in a premature stop codon (p.Leu71CysfsX33), resulting in a lack of the major catalytic domain, nuclear-localizing sequence, and C-terminal protein–protein interaction domain. This mutation frequency is reported in 2.5~6.7% of colorectal cancer and gastric cancer cases^37^.

### Chromosomal alterations

Various chromosomal alterations have also been discovered in different types of cancer (Table 1). *CARS1* fused to anaplastic lymphoma kinase (*ALK*) was identified in inflammatory myofibroblastic tumors (IMTs)^38^. Although *ALK* fusions with different partners are found in various cancers, the *CARS1-ALK* fusion has been reported only in IMT^39^. The CARS1-ALK fusion protein is predicted to produce an in-frame chimeric protein containing nearly 80% CARS1 at the N-terminus and a functional catalytic domain of ALK in the C-terminus. For this chromosomal rearrangement, CARS1 might contribute to neoplastic transformation by increasing the level of functional ALK because the fusion would provide the active *CARS1* promoter. Hence, it should be determined whether the CARS1-ALK fusion protein itself also contributes to tumorigenesis.

An oncogenic fusion protein translocated in liposarcoma (TLS) and CHOP (TLS-CHOP) is generated by a characteristic chromosomal translocation in myxoid and round cell liposarcomas^40^. As the genes encoding MARS1 and CHOP are located at the same position, i.e., 12q13, in opposite directions, they share a tail-to-tail overlap of approximately 55 base pairs in the 3ʹUTR. In particular, the overlap region contains the AU-rich regulatory element that controls mRNA stability. Thus, transcripts of *TLS-CHOP* and *MARS1* are hybridized together, and both mRNAs are stabilized. A reporter assay using the 3ʹUTR of *CHOP* WT and the AU-rich region deleted form showed reduced protein expression due to AU-rich region inhibition, indicating functional significance of the *MARS1* transcript for stabilizing the *TLS-CHOP* transcript. Because the *MARS1* mRNA would also be stabilized by the *TLS-CHOP* mRNA through the same mechanism, it would be interesting to determine whether the clinical significance of the TLS-CHOP fusion with regard to cancer relates to stabilization of the *MARS1* transcript in myxoid and round cell liposarcomas.

### Gene deletions

Locus 3p21.3 is frequently deleted in nasopharyngeal carcinoma. The *LARS2* gene is located at this locus, and complete deletion of the gene is observed in nasopharyngeal carcinoma^41^. Deletion of non-ARS genes also affects ARS expression. miR-15 and miR-16 are downregulated in chronic lymphocytic leukemia and pituitary adenoma, and frequent deletion of the loci 13q14 and 13q15 is observed, leading to the absence of miRNAs^42,43^. Interestingly, these miRNAs show 85% complimentary to the *RARS1* transcript, suggesting that RARS1 expression is regulated by miR-15 and miR-16. As expected, deletion of miR-15 and miR-16 increases the mRNA and protein levels of RARS1 in the above cancers^42^.

## ARS translation in cancer

Cancer-associated protein levels and posttranslational modifications (PTMs) of ARSs are reviewed in the following section.

### Control of ARS protein levels

**Fig. 3:**
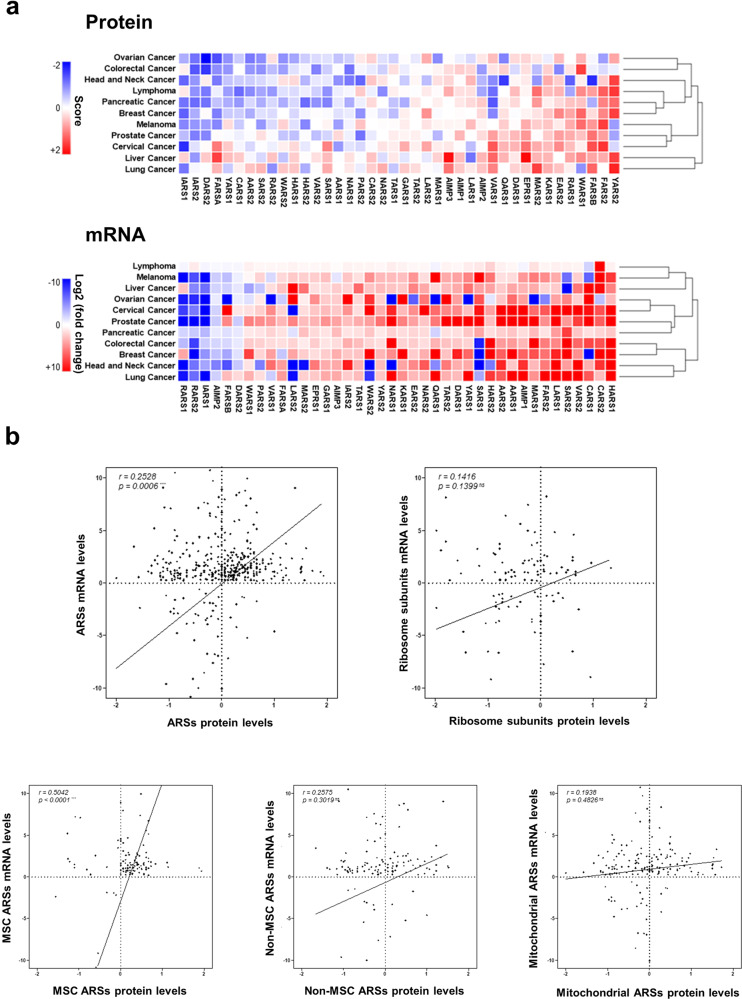
Comparison of cancer-associated ARS gene expression and protein levels. **a** The heatmap of cancer-associated ARS protein levels was generated with data extracted from The Human Protein Atlas (upper). The protein levels of each ARS are represented as a score that ranges from −2 to +2. The Human Protein Atlas provides information about protein levels based on the staining intensity in IHC images, which are classified into not detected, lowly detected, moderately detected and highly detected. We scored the staining intensities (not detected (−1), lowly detected (−1/3), moderately detected (+1/3) and highly detected (+1)) and averaged the score in normal and cancer tissues. Then, cancer-associated ARS protein levels were calculated by subtracting the normal score from the corresponding cancer score. A heatmap of the cancer-associated ARS mRNA levels was also generated with data extracted from TCGA (lower). The log2 of the fold change of each ARS mRNA in cancer tissue compared to normal tissue is displayed. Blue indicates low detection, red high detection, and white moderate detection in each cancer type. Cancer types and genes are hierarchically clustered based on the Pearson correlation score and average linkage (dendrogram shown for cancer types). **b** Correlation of the cancer-associated mRNA and protein levels of ARSs (top-left panel) and ribosome subunit proteins (top-right panel) is compared. The plot for ARSs is further divided into three different plots of MSC, non-MSC and mitochondrial ARSs (bottom panel). For analysis of ARSs, all 37 ARS types were investigated. For analysis of ribosome subunits, RPSA, RPS5, RPS6, RPS13, RPS20, RPLP0, RPL5, RLP8, RPL9, and RPL10A were investigated. Levels of protein and mRNA were calculated using the same method as in Fig. 4a. Cancer types shared by both databases were utilized for the correlation plot. The coefficient of correlation (r-value) and the significance level (p-value) were calculated via GraphPad PrismX. The coefficient of correlation and significance level were only statistically significant for ARSs.

Based on overall data from The Human Protein Atlas, different ARS protein levels in 11 different cancer types are displayed in the heatmap in Fig. 3a. IARS1 and IARS2 protein levels are reduced but those of YARS2 and FARS2 increased in most cancer types. In general, cytosolic ARSs show higher protein levels than mitochondrial ARSs in cancer^44^.

Higher levels of KARS1 protein are detected in breast cancer patient tissues^45^. A proteomic study of ARSs in LNCaP cells revealed androgen-dependent increases in AARS1, FARSA, GARS1, NARS1, TARS1, HARS1 and WARS1 levels. Further investigation of localized and metastatic prostate cancer and normal prostate tissues also showed elevated protein levels of GARS1 and KARS1 in cancer^12^. The catalytic activity of MARS1 is reported to be increased in colon cancer patient tissues, though protein levels were not directly compared^46^. Recently, the diagnostic value of MARS1 protein levels in bile duct cancer has been reported^47^. MARS1 exhibits stronger immunohistochemistry (IHC) staining signals in malignant biliary structures than in nonmalignant specimens^47^, and elevated LARS1 levels have been observed in 11 different types of non-small-cell lung cancer (NSCLC) and 12 colon cancer cell lines^10,48–50^. Moreover, clinical validation using NSCLC and colon cancer patient tissue showed higher LARS1 levels than normal tissue^51^. In contrast to LARS1, IHC using tissue microarrays of colorectal cancer revealed a negative correlation between WARS1 protein level and recurrence risk, lymph node metastasis and a more advanced stage, suggesting the prognostic value of the WARS1 protein level^23^.

To determine whether expression of ARS genes is tightly mirrored at the protein level, we compared a heatmap of cancer-associated ARS protein levels with that of ARS gene expression (Fig. 3a). We compared gene and protein levels of ARSs and other housekeeping translational components and ribosome subunits, including RPSA, RPS5, RPS6, RPS13, RPS20, RPLP0, RPL5, RLP8, RPL9, and RPL10A, and found a higher correlation value for ARSs than ribosomal subunits (Fig. 3b). We further analyzed the correlation value of subcategorized ARSs. ARSs are first categorized based on subcellular localization (cytosol and mitochondria); cytosolic ARSs are further categorized into multi-tRNA synthetase complex (MSC)-forming ARSs (DARS1, EPRS1, IARS1, KARS1, LARS1, MARS1, QARS1, RARS1, and AIMP1, 2, 3) and non-MSC ARSs (AARS1, CARS1, FARSA, FARSB, GARS1, HARS1, NARS1, SARS1, TARS1, VARS1, WARS1, YARS1). The plot of MSC ARSs shows a higher and statistically significant correlation value compared to that of non-MSC and mitochondrial ARSs (Fig. 3b). This feature suggests that ARSs, especially MSC components, can be used as reliable cancer biomarkers at both protein and gene transcription levels.

### Posttranslational modifications

Because ARS genes are constitutively expressed, their cellular activities, interaction, and cellular localization can be determined by signal- or stress-dependent specific PTMs. iPTMnet is a database that provides functional and structural analyses of posttranslational modifications^52^, showing 103 different PTMs of ARSs, with significance for KARS1, MARS1, EPRS1 and LARS1 PTMs in cancer cells, mouse models or even in patient samples.

**Fig. 4:**
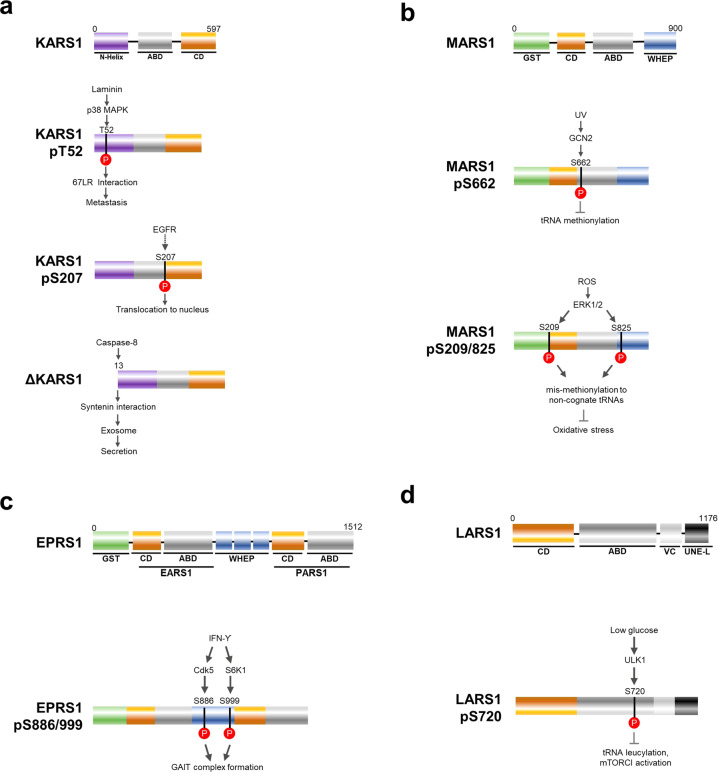
Posttranslational modification of ARSs in cancer. **a** Full-length KARS1 consists of the N-Helix, ABD and CD Domains. KARS1 is modified to three different forms by different kinds of upstream enzymes. First, KARS1 is phosphorylated at T52 by p38 MAPK in the presence of laminin. KARS1 pT52 is translocated to the plasma membrane for interaction with 67LR and then promotes metastasis. Second, KARS1 is phosphorylated at S207 upon EGFR signaling pathway activation. KARS1 pS207 is translocated to the nucleus and appears to be associated with disease-free survival of NSCLC. Third, the N-terminal 12 amino acid KARS1 is cleaved by caspase-8 to produce ΔKARS1. ΔKARS1 is secreted into the extracellular space in exosomes via interaction with syntenin. **b** Full-length MARS1 consists of GST, CD, ABD and WHEP domains. MARS1 is modified to two different forms of MARS1 in response to various input signals. Upon UV irradiation, MARS1 is phosphorylated at S662 by GCN2. MARS1 pS662 has a decreased capability to methionylate tRNAs, resulting in downregulation of global translation. In contrast, MARS1 is doubly phosphorylated at S209 and S825 by ERK1/2 in response to oxidative stress. MARS1 pS209/825 shows increased mismethionylation to noncognate tRNAs due to an increase in Met residues in proteins, which contribute to reducing ROS levels. **c** Upon stimulation with IFN-γ, EPRS1 is phosphorylated at S886 and S999 by CDK5 and S6K1, respectively. EPRS1 pS886/999 forms the GAIT complex to regulate translation of GAIT elements. **d** LARS1 is phosphorylated at S720 by ULK1 in response to glucose starvation. LARS1 S720 shows decreased leucine binding capability, resulting in decreased tRNA leucylation and mTORC1 stimulation. CD, catalytic domain; ABD, anticodon-binding domain; ROS, reactive oxygen species.

KARS1 is phosphorylated at two distinct residues in response to different signals and modulates cancer-associated characteristics (Fig. 4a). First, KARS1 is phosphorylated at T52 by p38MAPK in the presence of laminin. Phosphorylated KARS1 dissociates from MSCs and is translocated to the plasma membrane for interaction with the 67-kDa laminin receptor (67LR). KARS1 stabilizes 67LR, leading to increased cell migration and cancer metastasis^53^. In contrast, KARS1 is phosphorylated at S207 following activation of the EGFR signaling pathway, which appears to predict disease-free survival of NSCLC^54^. In addition, N-terminal cleavage of KARS1 occurs in colorectal cancer cell lines. Upon serum starvation, the N-terminal 12 amino acid peptide of KARS1 is cleaved by caspase-8, causing its dissociation from MSCs. Cleaved KARS1 interacts with syntenin for exosome biogenesis and is then secreted via exosomes to trigger macrophage/neutrophil migration and inflammation^55^. Interestingly, a recent study reported that colorectal cancer patients have higher KARS1 levels in plasma^56^. In general, it is worth monitoring whether plasma KARS1 is carried by circulating exosomes or as a naked form.

MARS1 is also phosphorylated in response to two distinct signals (Fig. 4b). Upon UV irradiation, general control nonrepressed-2 (GCN2) phosphorylates MARS1 at S662, decreasing tRNA^Met^ methionylation activity and releasing bound AIMP3, which then translocates to the nucleus for DNA repair^57^. Considering that UV irradiation is a carcinogenic stimulus and AIMP3 is a tumor suppressor maintaining genomic stability^58,59^, dysregulated phosphorylation of MARS1 at S662 may be associated with cancer development. MARS1 is also phosphorylated at S209 and S825 by extracellular signal-related kinase (ERK1/2) in response to oxidative stress. Double-phosphorylated MARS1 exhibits decreased specificity for tRNA^Met^ and charges methionine to nonmethionyl tRNAs, resulting in more frequent methionine incorporation into nascent proteins, which increases cellular reductive capacity^60^. Cells expressing phosphorylation-deficient mutants are more sensitive to oxidative stresses, suggesting that these cells defend against oxidative stress by utilizing S209/S825 phosphorylated MARS1 to promiscuously charge methionine to many different tRNAs. This mechanism may also be functionally related to the reactive oxygen species (ROS)-managing mechanism in cancer.

As mentioned above, EPRS1 forms the GAIT complex in the presence of IFN-γ to regulate GAIT element-containing mRNAs, including *VEGFA*. Two-step phosphorylation at S886 and S999 mediated by cyclin-dependent kinase 5 (CDK5) and ribosomal protein S6 kinase beta-1 (S6K1), respectively, is necessary for EPRS1 to dissociate from MSCs and subsequently associate with the GAIT complex (Fig. 4c)^61,62^. pS886 is required for interaction with NSAP1, L13a and GAPDH, and pS999 directs binding to eIF4G for formation of the functional GAIT complex.

Posttranslational modification of LARS1 was recently identified. Under glucose deprivation, Unc-51-like autophagy activating kinase 1 (ULK1) phosphorylates LARS1 at S720 (Fig. 4d). This modification is reported to decrease leucine binding affinity, inhibiting tRNA^Leu^ leucylation and mechanistic target of rapamycin complex 1 (mTORC1)-stimulating activities to save energy. In addition, cells expressing phosphomimetic mutants show increased leucine degradation for energy generation in rhabdomyosarcoma cell lines^63^. Although the exact role of LARS1 phosphorylation at S720 in cancer is not fully understood, this work suggest that LARS1 modulates its leucine binding capability under metabolic stress in cancer, such as glucose starvation, thereby providing a metabolic adaptation and survival strategy.

## Cancer-associated functions

**Fig. 5:**
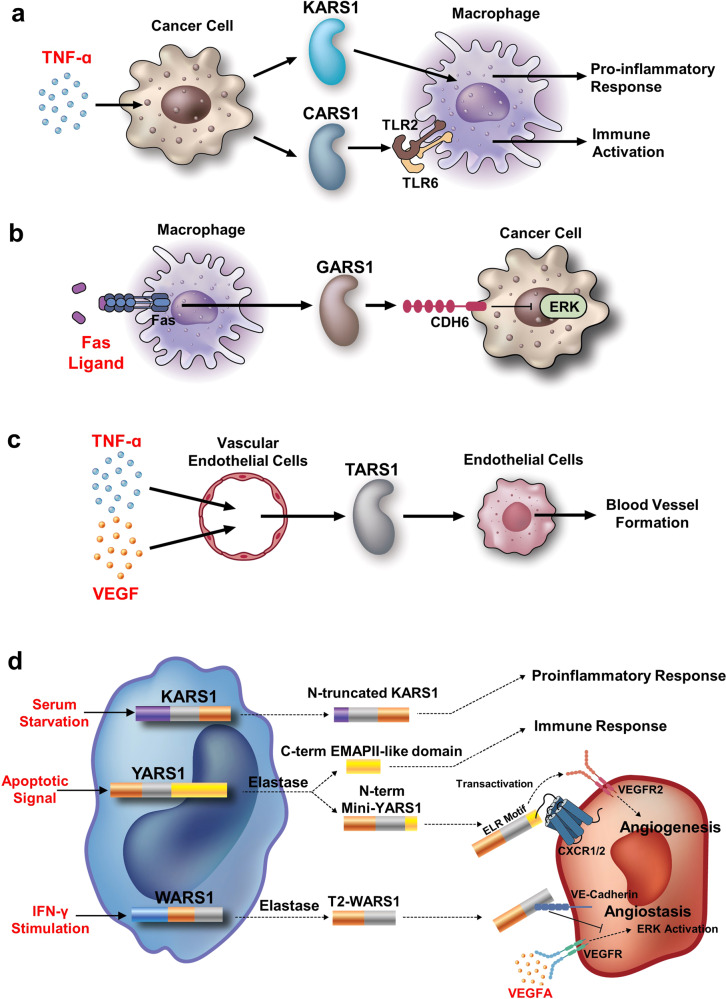
Secreted ARSs function in cancer. **a** KARS1 and CARS1 are secreted from cancer cells by TNF-α signaling to induce immune responses via macrophages. CARS1 appears to function via TLR2/6; KARS1’s functional receptor has yet to be identified. **b** Upon stimulation with Fas, GARS1 is secreted from macrophages and induces cancer cell death via CCDH6. Binding of GARS1 to CDH6 releases PP2A from CDH6 to deactivate the ERK signaling pathway required for cancer cell survival. **c** Vascular endothelial cells secrete TARS1 upon TNF-α or VEGF stimulation, promoting blood vessel formation. **d** Proteolytic cleavage of ARSs induces secretion or activation of their extracellular activities. The N-truncated KARS1 generated by caspase-8 is secreted upon serum starvation to induce a proinflammatory response. Upon apoptotic signaling, YARS1 is secreted and cleaved via elastase to produce the C-terminal EMAP II-like domain and N-terminal mini-YARS1. The EMAP II-like domain activates the immune response; mini-YARS1 binds to CXCR1/2 through its ELR motif for angiogenesis. Elastase cleaves WARS1 to produce T2-WARS1 upon IFN-ɣ stimulation. T2-WARS1 binds to VE-cadherin, leading to inhibition of VEGFA-activated VEGFR signal transduction.

**Fig. 6:**
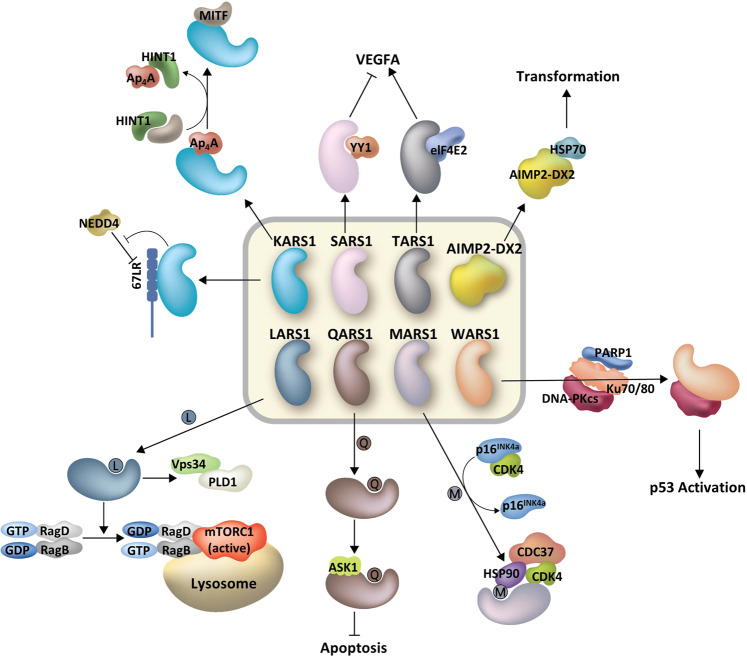
Intracellular signaling functions of ARSs via diverse protein–protein interactions. ARSs mediate diverse intracellular and extracellular signaling pathways by protein–protein interactions, some of which are further controlled by amino acid sensing and by generating second messengers such as diadenosine polyphosphates. KARS1 inhibits NEDD4 and stabilizes 67LR through its interaction with 67LR. KARS1-mediated Ap_4_A production increases the transcriptional activity of MITF by liberating HINT1. SARS1 inhibits VEGFA transcription through its interaction with YY1; the TARS1-eIF4E2 complex increases translation initiation of VEGFA. Oncogenic AIMP2-DX2 is stabilized by its association with HSP70, leading to cell transformation. LARS1 mediates mTORC1 activation through leucine-dependent interactions with either RagD or Vps34. QARS1 decreases apoptosis through glutamine-dependent interactions with ASK1. MARS1 competes with p16^INK4a^ for interaction with CDK4. Interaction between MARS1 and CDK4 is possibly dependent on methionine, and the MARS1-CDK4-HSP90-CDC37 complex increases the stability of CDK4. WARS1 mediates PARylation of DNA-PKcs, leading to p53 activation. Association of WARS1, PARP-1, and DNA-PKcs might also be dependent on tryptophan.

In addition to a catalytic role in translation to meet the increased demand for protein synthesis for cancer cell growth, ARSs can be involved in the processes of tumorigenesis in multiple ways. First, ARSs play unique roles in the extracellular space (Fig. 5). Second, ARSs mediate a broad spectrum of cellular signaling pathways via specific protein–protein interactions with diverse cellular factors (Fig. 6, upper). Third, ARSs control their catalytic and signaling activities in an amino acid-dependent manner (Fig. 6, lower). Fourth, ARSs generate the second messenger molecules diadenosine polyphosphates (Ap_n_As). Among their diverse regulatory activities, this section below focuses on the functions of ARSs associated with cancer development and maintenance.

### Secretory functions

Although ARSs primarily operate in intracellular locations, they are known to mediate cell-to-cell communication. Indeed, they are secreted from cells such as macrophages and cancer cells as naïve or modified forms in response to specific environmental stimuli and exert unique extracellular activities (Fig. 5).

For instance, TNF-α induces secretion of KARS1 from cancer cells to the extracellular space (Fig. 5a). Although the specific mechanism by which TNF-α mediates KARS1 secretion is not fully understood, it was reported that secreted KARS1 stimulates proinflammatory responses when it acts on immune cells such as macrophages^45^. Cancer cells are also capable of secreting CARS1 upon stimulatory signals such as TNF-α and the ER stress inducer tunicamycin (Fig. 5a)^64^. Secreted CARS1 activates immune responses by directly interacting with toll-like receptor 2 (TLR2) on antigen-presenting cells, including dendritic cells. UNE-C1 in the catalytic domain of CARS1 was determined to serve as the binding domain for TLR2. The CARS1 UNE-C1 domain shows a synergistic effect with cancer antigens and several immune checkpoint inhibitors in in vivo cancer models, suggesting further potential for the domain to be developed as an immunoadjuvant to activate antitumor immunity.

In contrast, GARS1 is secreted from macrophages when stimulated by Fas ligand (Fig. 5b). Secreted GARS1 interacts with cadherin 6 (CDH6) on neighboring cancer cells, leading to phosphatase 2 A (PP2A) release, which deactivates the ERK signaling pathway^19^. Administration of purified recombinant GARS1 induces tumor regression in vivo in tumor models^19^, suggesting its potential as a novel anticancer agent.

After the discovery of autoantibodies specific for TARS1 in human sera^65^, TARS1 was predicted to be secreted from cells, and the role of secreted TARS1 was confirmed in blood vessel formation (Fig. 5c)^66^. After VEGF or TNF-α stimulation, TARS1 is secreted from human endothelial cells. TARS1 treatment of endothelial cells, fertilized chicken embryos and zebrafish increases blood vessel formation in different in vitro and in vivo models^66,67^. Further investigation of the exact mechanism by which secreted TARS1 increases angiogenesis would provide functional insight into the roles of other ARSs, including YARS1 and WARS1, in tumor-associated vascularization.

Proteolytic cleavages also appear to be involved in the secretion or proper extracellular function of some ARSs (Fig. 5d). As discussed above, serum starvation triggers N-terminal truncation of KARS1 via activated caspase-8, leading to exosome-mediated secretion from cancer cells for proinflammatory activities^56,68^. In response to apoptotic signals, YARS1 is secreted and then cleaved by elastase to generate N-terminal mini-YARS1 and C-terminal EMAPII-like domains. The C-terminal EMAPII-like domain increases the migration of mononuclear phagocytes and TNF-α production, and N-terminal mini-YARS1 induces the migration of polymorphonuclear leukocytes and HUVECs and angiogenesis^69–71^. Mini-YARS1, but not full-length YARS1, has such activities because cleavage of the C-terminal EMAPII-like domain enables exposure of the ELR motif of mini-YARS1, through which it binds to C-X-C motif chemokine receptor 1/2 (CXCR1/2)^72^. By binding to CXCR1/2, mini-YARS1 appears to transactivate VEGF receptor 2 (VEGFR2) as well as downstream angiogenesis-associated signaling molecules for blood vessel formation^70^. In contrast to YARS1, truncated forms of WARS1 exhibit angiostatic function. Upon stimulation with IFN-γ, either full-length or mini-WARS1, the alternative splicing form of WARS1, as described above, is secreted from cells. In the extracellular space, the N-terminal end of WARS1 is digested by elastase to produce the T1 and T2 forms of the protein^73,74^. Similar to YARS1, only truncated forms, but not full-length WARS1, show angiostatic activity. T2-WARS1 binds to VE-cadherin on endothelial cells through interaction between the tryptophan residue of VE-cadherin and the catalytic pocket of WARS1^75^. Binding of T2-WARS1 to VE-cadherin decreases VEGF-mediated activation of the ERK pathway, suggesting that its antiangiogenic function is mediated by VE-cadherin^76^.

### Protein–protein interactions

ARSs also have diverse regulatory activities via interactions with different cellular factors^77^. The functional ARS interactome broadly covers major signaling pathways to control cellular growth, proliferation and death, crucially influencing the process of tumorigenesis (Fig. 6)^78^. SARS1 and TARS1 control VEGFA levels at different stages via interactions with their specific partners^79,80^. SARS1 interacts with the transcription factor YY1 and then represses transcriptional activation of VEGFA. Interaction between SARS1 and YY1 seems to be crucial for repression of VEGFA transcription, as zebrafish with knockdown of either SARS1 or YY1 develop more intersegmental vessels^79^. TARS1 regulates translation of some mRNAs, including VEGFA, through the formation of a novel translation initiation complex composed of TARS1 and eukaryotic translation initiation factor 4E family member 2 (eIF4E2). The TARS1 and eIF4E2 complex successfully initiates translation of some mRNAs that are selected via the specific RNA-binding ability of TARS1 and the cap-binding ability of eIF4E2^80^. Although the functional significance of VEGFA regulation in cancer was not directly examined in either study, dysregulated interaction of the two factors may affect cancer growth via VEGF expression.

AIMP2-DX2 is a splicing variant of AIMP2 lacking exon 2 and is known to disturb the tumor-suppressive activities of AIMP2 through competitive interaction with target proteins^81,82^. Overexpression of AIMP2-DX2 correlates positively with cancer progression, and a recent study reported that AIMP2-DX2 is stabilized through interaction with heat shock protein 70 (HSP70)^83^. HSP70 binding to AIMP2-DX2 appears to prevent the association of Siah E3 ubiquitin protein ligase 1 (Siah1) with AIMP2-DX2, reducing AIMP2-DX2 degradation. Chemical inhibition of AIMP2-DX2 and HSP70 interaction successfully decreases AIMP2-DX2 levels in cells and induces tumor regression in an in vivo mouse model, suggesting the interface of AIMP2-DX2 and HSP70 as a novel target to control cancer.

EPRS1 forms the GAIT complex to inhibit the proinflammatory response. KARS1 interacts with 67LR to promote cell migration. As mentioned above, specific interactions between secreted ARSs and specific receptors have been reported, including the pairs GARS1-CDH6^19^, CARS1-TLR2^64^, YARS1-CXCR1/2^70^ and T2-WARS1-VE-cadherin^75^. These interactions may be positively or negatively implicated in cancer development.

### Intracellular amino acid sensory activities

After the functional significance of amino acids as signaling molecules was suggested, cellular amino acid sensors have attracted much attention^84–88^. As ARSs specifically recognize corresponding amino acids for their catalytic activities, they have intrinsic potential to sense the intracellular levels of amino acids. For instance, LARS1 senses intracellular leucine levels to activate the mTORC1 pathway (Fig. 6)^84^. Mechanistically, leucine-bound LARS1 is translocated to lysosomes and interacts with RagD GTPase. Through conversion of RagD-GTP to RagD-GDP, mTORC1 is recruited to lysosomes and activated, promoting cell proliferation and growth. In this context, LARS1 functions as a GTPase-activating protein (GAP) in the Rag GTPase cycle^89^ in coordination with other leucine sensors, such as Sestrin2^85^. The role of LARS1 in the mTORC1 pathway was also shown in the axis of vacuolar protein sorting 34 (Vps34)-phospholipase D1 (PLD1)^90^. Leucine-bound LARS1 activates Vps34, accumulating phosphatidylinositol 3-phosphate (PI-3-P) for PLD1 activation. Activated PLD1 is recruited to lysosomes and generates phosphatidic acid (PA) for activation of mTORC1. Overall, LARS1 appears to control the activity of mTORC1 through multiple pathways in a leucine-dependent manner^84,90,91^. The functional significance of LARS1 as a leucine sensor for mTORC1 activation in cancer has been further investigated in different cancer cell lines and in vivo models. Chemical inhibition of the interaction between LARS1 and RagD decreases the proliferation and increases the death of colon and lung cancer cell lines but not normal cell lines. In addition, a chemical inhibitor induces tumor regression in a mouse xenograft model using colon and lung cancer cell lines, even though the cells show rapamycin resistance^51,92^. Considering that LARS1 is overexpressed in some cancers, including myeloid leukemia^93^, pancreatic cancer, renal cancer, cervical cancer and skin cancer (Fig. 3), targeting the leucine-sensing-mediated function of LARS1 has therapeutic potential against cancer.

Glutamine is one of the most crucial amino acids in tumor progression. Glutamine depletion induces apoptosis, whereas glutamine supplementation protects cells through various molecular pathways^94,95^. Interestingly, QARS1 was shown to mediate the antiapoptotic property of glutamine^96^. Mechanistically, QARS1 forms a protein complex with apoptosis signal-regulating kinase 1 (ASK1) in a glutamine-dependent manner. In the presence of glutamine, QARS1 and ASK1 interact through their C-terminal domains, decreasing the kinase activity of ASK1 for apoptosis. MARS1 was previously shown to stabilize cyclin-dependent kinase 4 (CDK4)^97^, which forms a complex with cyclin D1 and regulates the cell cycle transition from G1 to S phase^98^. MARS1 contributes to proper folding of CDK4, along with heat shock protein 90 (HSP90) and cell division cycle 37 (CDC37), which then interacts with cyclin D1^99^. Although direct evidence for methionine-mediated interaction between MARS1 and CDK4 was not provided, methionine binding-deficient mutants of MARS1 and a methionine analog, FSMO, reduced interaction with CDK4, implying that interaction between these two proteins may be sensitive to the binding status of methionine to MARS1. p16^INK4a^ is a tumor suppressor that negatively regulates the CDK4 and cyclin D1 complex, which activates oncogenes such as Rb and E2F^98,100^. The effect of MARS1-mediated CDK4 stabilization is more prominent in p16^INK4a^-negative cancers because MARS1 and p16^INK4a^ appear to compete for interaction with CDK4. Indeed, p16^INK4a^-negative cancer cell lines show a higher positive correlation for MARS1 and CDK4 protein levels than p16^INK4a^-positive cancer cell lines. Thus, targeting MARS1 using methionine analogs may be an attractive way to control p16^INK4a^-negative cancer via CDK4.

WARS1-mediated poly(ADP-ribosy)lation (PARylation) of the DNA-dependent protein kinase catalytic subunit (DNA-PKcs) also exhibits a potential connection with amino acid binding^101^. After IFN-γ stimulation, the WARS1 protein level increases, and at least some of this increased WARS1 population plays a role in activating DNA-PKcs and p53 through DNA-PKcs PARlyation. Although the effect of tryptophan on the formation of WARS1, poly(ADP-ribose) polymerase-1 (PARP-1) and the DNA-PKcs complex has not been clearly elucidated, 5’-O-[N-(9 L-tryptophanyl) sulfamoyl] adenosine (Trp-SA), a Trp-AMP analog, dissociates this triple complex; thus, catalytic site occupation might be crucial for this function. Overall, the functional significance of Trp or Trp-SA analogs on IFN-γ-induced cancer cell death should be further investigated.

### Diadenosine polyphosphate-producing activities

Some ARSs are known to produce second-messenger molecules (Ap_n_A), especially diadenosine triphosphate (Ap_3_A) and diadenosine tetraphosphate (Ap_4_A). Because Ap_n_A is produced when the reaction intermediate AA-AMP reacts with additional ATP molecules, low cognate tRNA levels can increase Ap_n_A formation^102^. After the synthesis of Ap_4_A was first discovered in an in vitro system with purified ARS, amino acids, ATP and Mg^2+^, this reaction was verified in vivo^103^. Their function as secondary messengers that amplify downstream signaling pathways appears to be involved in cancer^102^.

KARS1 controls the activity of microphthalmia-associated transcription factor (MITF) by producing the Ap_4_A molecule (Fig. 6)^104^. When KARS1 is released from the MSC, it forms a protein complex with MITF and histidine triad nucleotide-binding protein 1 (HINT1) inside the nucleus. In quiescent cells, MITF interacts with HINT1, which represses the transcriptional activity of MITF. However, the Ap_4_A second messenger molecule is produced by KARS1, and it binds to HINT1 such that MITF is liberated for activation of target gene transcription. Although the function of KARS1-mediated MITF activation has mainly been elucidated in immune cells, this mechanism might also be important for tumor development^54^.

## Concluding remarks

**Fig. 7:**
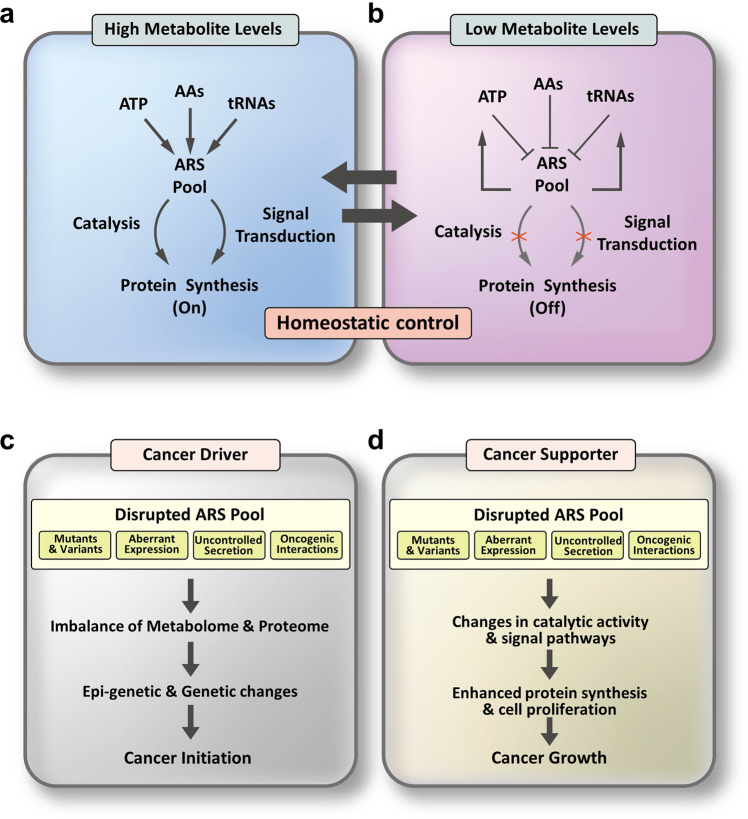
Potential role of ARSs in the pathogenesis of cancer. **a**, **b** ARSs may regulate protein synthesis by integrating information about metabolite levels. In cells with high metabolite levels, ARSs increase protein synthesis through catalytic and signal transduction pathways; under low metabolite conditions, ARSs decrease protein synthesis by inhibiting catalytic and signal transduction pathways to achieve metabolite balance through metabolic reprogramming. **c**, **d** ARS may function as either a driver or supporter in tumor development. Disruption of the ARS pool by mutation, aberrant expression, uncontrolled secretion and oncogenic interaction results in an imbalance of the metabolome and proteome, which can trigger epigenetic and genetic changes for cancer initiation. In addition, a disrupted ARS pool supports cancer growth by enhancing protein synthesis and cell proliferation.

ARSs utilize amino acids as reaction substrates, consuming ATP as an energy source and the cellular tRNA pool as the vehicle to carry charged amino acids to ribosomes. Thus, metabolic balance among amino acids in coordination with ATP and tRNAs is crucial for the protein synthesis rate and fidelity. When intracellular levels of the reaction substrates are high, ARSs can enhance protein synthesis via dual pathways of catalysis and signal transduction (Fig. 7a). In cells with lower levels of amino acids, ATP and tRNAs, ARSs may reduce or stop protein synthesis and function to reprogram metabolism to return amino acid, ATP and tRNA levels to normal (Fig. 7b). Thus, the cellular pool of ARSs contributes to the homeostasis of the cellular metabolome and proteome not only via their catalytic activities but also via their multifarious regulatory capability.

As described above, multiple genomic and transcriptomic analyses show that ARS-encoding genes are specifically under- or overexpressed in different types of cancer cell lines. Cancer-associated genetic alterations of ARSs, including SNPs, splicing variants, single mutations, and deletions, have also been found. In addition, cancer-related PTMs and secretion of ARSs have been reported. Considering the diversity of cancer-associated changes in ARS-encoding genes and proteins, ARSs may be involved in cancer formation in a systemic manner rather than through the participation of only a few of them. Regardless of the cancer-associated changes in ARSs at the gene and protein levels, these changes would ultimately affect homeostatic control of the metabolome (particularly related to amino acids, energy and RNAs). In theory, ARSs can function as cancer drivers or supporters. As a cancer driver, a disrupted ARS pool would cause an imbalance of the metabolome and proteome, resulting epigenetic and genetic changes and eventually provoking cancer (Fig. 7c). Alternatively, enhanced expression or mutations and aberrant forms of ARSs can increase their catalytic activities and signaling pathways to support the increased demand for protein synthesis required for cancer cell growth (Fig. 7d).

From a therapeutic point of view, diverse biological activities of human ARSs indicate their potential as therapeutic targets and agents for cancer treatment^1^. For instance, inhibition of the catalytic site^105^ and noncatalytic site of LARS1 responsible for interaction with RagD have been shown to be effective in controlling the tumor-promoting mTORC1 pathway^92^. Furthermore, chemical intervention of interaction between KARS1 and 67LR in the cell membrane is effective against cancer metastasis^53^. Targeting a splicing variant of AIMP2 at the interface with HSP70 also effectively suppresses tumor growth^83^. Secreted GARS1 and CARS1 exhibit potent anticancer activities via their specific and unique modes of action^64^.

The potential of ARSs as therapeutic targets has not been seriously explored because they are essential enzymes for protein synthesis, with concern for a general effect on the body. Nevertheless, recent unexpected discoveries regarding their specific roles in diverse regulatory pathways are rapidly opening a new possibility for ARSs as druggable target families. First, global protein synthesis is not much affected, even when cellular expression of ARSs is significantly suppressed or their catalytic activities are inhibited. Perhaps cellular levels of ARSs are higher than those required to meet the demand of global protein synthesis for highly differentiated normal cells. Thus, even if a cellular ARS is crippled by transcriptional suppression or catalytic inhibition, it may not seriously affect global protein synthesis and cell viability, as expected. Second, only a small portion of cellular ARSs is actually used for their epi-translational activities as exerted by the extracellular space, cell membrane and nucleus. Thus, targeting ARSs with regard to these activities would specifically modulate pathologically relevant activities while not affecting global protein synthesis. In general, specific targeting of a portion of ARSs that are involved in epitranslational activities might show highly specific and potent efficacy toward pathological phenotypes of diseases. Third, the diverse-yet-idiosyncratic activities of ARSs provide multiple options for developing drugs not only for cancer but also for other refractory diseases with no effective drugs available.
